# Overdominant expression of related genes of ion homeostasis improves K^+^ content advantage in hybrid tobacco leaves

**DOI:** 10.1186/s12870-022-03719-1

**Published:** 2022-07-12

**Authors:** Kai Pi, Wen Luo, Zejun Mo, Lili Duan, Yuzhou Ke, Pingsong Wang, Shuaibo Zeng, Yin Huang, Renxiang Liu

**Affiliations:** 1 College of Tobacco, Guizhou University, Huaxi District, Guizhou Province 550025 Guiyang City, P. R. China; 2 Key Laboratory for Tobacco Quality Research Guizhou Province, Guizhou University, 550025 Guiyang, P. R. China; 3 College of Agriculture, Guizhou University, 550025 Guiyang, P. R. China

**Keywords:** Heterosis, Tobacco, Potassium, Ion homeostasis

## Abstract

**Background:**

Potassium(K^+^) plays a vital role in improving the quality of tobacco leaves. However, how to improve the potassium content of tobacco leaves has always been a difficult problem in tobacco planting. K^+^ content in tobacco hybrid is characterized by heterosis, which can improve the quality of tobacco leaves, but its underlying molecular genetic mechanisms remain unclear.

**Results:**

Through a two-year field experiment, G70×GDH11 with strong heterosis and K326×GDH11 with weak heterosis were screened out. Transcriptome analyses revealed that 80.89% and 57.28% of the differentially expressed genes (DEGs) in the strong and weak heterosis combinations exhibited an overdominant expression pattern, respectively. The genes that up-regulated the overdominant expression in the strong heterosis hybrids were significantly enriched in the ion homeostasis. Genes involved in K^+^ transport *(KAT1/2, GORK, AKT2, and KEA3*), activity regulation complex (*CBL-CIPK5/6*), and vacuole (*TPKs*) genes were overdominant expressed in strong heterosis hybrids, which contributed to K^+^ homeostasis and heterosis in tobacco leaves.

**Conclusions:**

K^+^ homeostasis and accumulation in tobacco hybrids were collectively improved. The overdominant expression of K^+^ transport and homeostasis-related genes conducted a crucial role in the heterosis of K^+^ content in tobacco leaves.

**Supplementary Information:**

The online version contains supplementary material available at 10.1186/s12870-022-03719-1.

## Background

Heterosis is a common biological phenomenon in nature. Heterosis refers to the phenomenon in which the F1 heterozygote produced through hybridization is superior in growth potential, biomass, stress resistance, and adaptability than the parents. Shull used the term “heterosis” in plant breeding for the first time [1]. In the last century, East pointed out that heterosis was formed by hybridizing different allotetraploids in the same tobacco genus [2], which Darwin observed as well in earlier times [3]. Heterosis is a complex biological phenomenon influenced by multiple factors. Many scholars put forward several hypotheses to analyze the genetic basis of heterosis, mainly embodied in three hypotheses: The dominance [4, 5], overdominance [2], and epistasis.

K^+^, an essential element for plant growth and development, is present mainly in metabolically active parts of the plant in the ionic state [6]. Wang and Wu revealed that K^+^ influences plant yield, quality, and stress resistance [7] through its involvement in protein synthesis, cellular osmoregulation, photosynthesis, stomatal movement, and enzyme activation [8]. Moreover, we were informed that K^+^ was crucial for tobacco leaf quality, which could significantly improve tobacco leaves’ color, combustion force, and fire maintenance power, moderate tobacco leaves’ identity, and increase their elasticity and softness [9]. The potassium content of tobacco leaves changes abruptly during the topping period and decreases rapidly after topping, resulting in a low potassium content in mature tobacco leaves, which is the focus of research on tobacco quality [9]. Potassium fertilizer application is a common technology to improve crop yield and quality, but the high input of K^+^ fertilizer and low utilization rate increase the production cost and cause environmental problems [10, 11]. Therefore, it is crucial to improve the utilization efficiency of K^+^ in tobacco and to select potassium-efficient tobacco germplasm [12].

It has been reported that K^+^ homeostasis is crucial for plant growth and environmental adaptation [13]. Specifically, it allows plants to survive salt stress [14]. It is worth noting that ion homeostasis maintenance requires the precise regulation of various ion transport proteins, including transporter pumps, carriers, and ion channels. Additionally, tobacco plants with highly expressed *AtHKT1* could maintain K^+^ homeostasis under high Na + stress [15]. Furthermore, under such a condition, hybrids can maintain ion homeostasis via mediating sodium and potassium transport proteins [16], and the transport of K^+^ between vacuoles and cytoplasm contributes to K^+^ homeostasis [17].

K^+^ channel is a crucial pathway for potassium uptake in tobacco, facilitating direct uptake of potassium from the soil solution, regulating the transport of potassium from the roots to the leaves, and coordinating the accumulation and homeostasis of K^+^ in tobacco leaves [18]. Many genes involved in the absorption and transportation of potassium, including the potassium channel gene and potassium ion transporter gene, have been identified in various crops, which play different roles in potassium absorption, transportation, and homeostasis [19]. In leaf cells, *KAT1/2* and *GORK* mediate K^+^ inflow and outflow through the plasma membrane, respectively [20]. Weak inward rectification channel *AKT2* mediates K^+^ entry into the phloem [21], and its activity is regulated by the *CBL4-CIPK6* complex [6, 22]. *NHX1/2* transports K^+^ into vacuoles, establishing vacuole potassium pool and homeostasis [23–25]. The tandem pore channels *TPKs* and *TPCs* mediate the efflux of K^+^ from the vesicles into the cytoplasm [26, 27]. The channel activity of *TPKs* is regulated by the *CBL-CIPK6* complex [28]. K^+^ efflux trans-transporter *KEA1/2/3* mediates K^+^ transport in chloroplasts and promotes photosynthesis [29, 30]. *KEA4/5/6* transports K^+^ from the cytoplasm to the inner lumen of the Golgi apparatus, the Golgi network, and the anterior chamber/multivesicular body, functioning in the endosomal network to maintain pH [31].

*Nicotiana tabacum* L. is a typical allotetraploid crop, a natural cross of two ancestors, *Nicotiana sylvestris* and *Nicotiana tomentosiformis* [32]. Hybrid dominance in intra- and interspecific tobacco varieties is prominent [33–35]. Heterosis has been widely studied for excellent performance in growth capacity, leaf yield, quality, plant height, and disease resistance of tobacco verities [34, 36–38]. In our research on the hybrid selection based on the heterosis, we found a clear heterosis advantage in the potassium content of tobacco leaves, and tobacco varieties with higher K^+^ content could be obtained through hybridization [39]. However, the reasons for the heterosis of K^+^ content in tobacco leaves must be further clarified. Therefore, the current study analyzed the molecular mechanism underlying the formation of heterosis of K^+^ content in tobacco leaves at the transcription level.

## Results

### Heterosis performance of K^+^ content in leaves of F1 hybrids

**Fig. 1:**
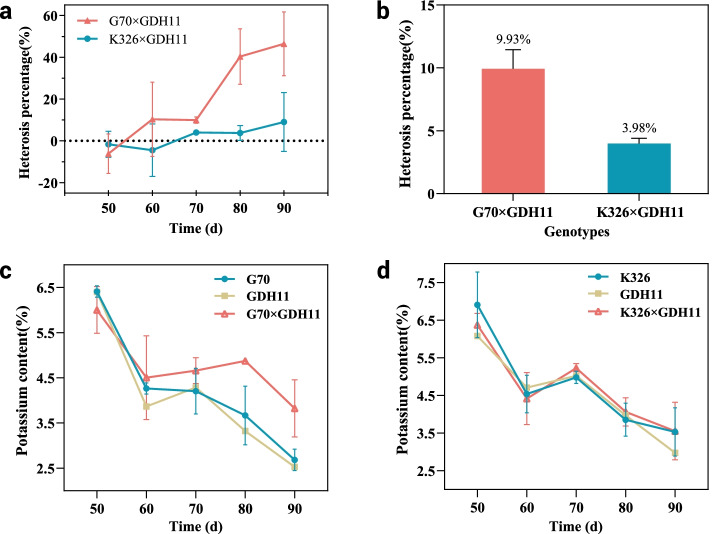
Heterosis and variation trend of K^+^ content in tobacco leaves. **a** shows the change trend of heterosis of K^+^ content of F_1_ hybrid during tobacco leaf growth. **b** shows the heterosis value of H and l hybrids at 70 days after transplanting. **c** and **d** respectively represent the change trend of K^+^ content of H and L hybrids and their parents after 50 ~ 90 days of transplantation

Samples were taken every 10 days from days 50 to 90 after transplantation to determine the K^+^ content of parents and their hybrids (Additional file 1). The heterosis value of K^+^ content was determined by analyzing the K^+^ content of leaves in different hybrid combinations, revealing that most hybrids did not exhibit heterosis from day 50 to 60. In contrast, the heterosis of K^+^ content changed the most from day 70 to 80 (Fig. 1a). To explore the causes of heterosis in K^+^ content, we used fresh tobacco samples 70 days after transplantation for transcriptome sequencing analysis. During this period, Heterosis of K^+^ content was the highest in hybrid G70×GDH11(H) (9.93%), and that of hybrid K326×GDH11 (L) was the weakest (3.98%) (Fig. 1b). H hybrids did not reveal a significant hybrid advantage from day 50 to 60 (Fig. 1c). On the contrary, the K^+^ content of the L hybrid and its parents demonstrated a downward trend 70 days later (Fig. 1d). Therefore, we selected strong heterosis hybrid G70×GDH11(H), weak heterosis hybrid K326×GDH11(L), and their parents to explore the reasons for the formation of K^+^ content heterosis.

### Transcriptome differences of leaves between parents and F_1_ hybrids

We used two hybrids with different hybridization advantages and their parents as sequencing materials to construct 18 cDNA libraries and then sequenced them on NovaSeq 6000 platform. The average error rate of all samples was 0.02% (Q20 > 98%, Q30 > 94%, and GC content > 43%) (Additional file 2). The clean sequence accounted for more than 99% of the total sequence (Additional file 3). The results revealed that the sequencing data was reliable and used for further analyses.

**Fig. 2:**
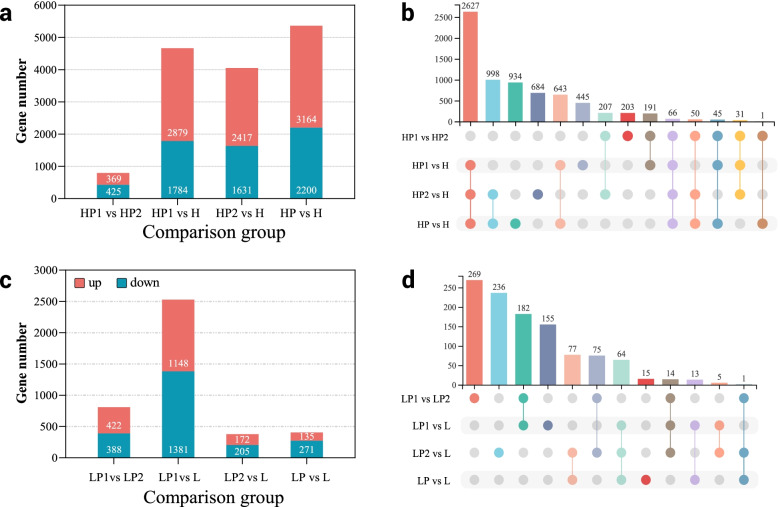
Total DEGs and distribution in hybrids and their parents. **a** and **b** respectively represent the total number of DEGs in H and L hybrids and their parents. **c** and **d** respectively represent the common differential genes of DEGs in each comparison group

To discover the reasons for K^+^ content heterosis at the transcriptome level, DESeq2 software was used to compare the DEGs between hybrid and its parents. The DEGs between the two parents were also analyzed. At *P* ≤ 0.05 and |log2 Fold Change| ≥ 1, we identified that 2879 genes were up-regulated and 1784 genes were down-regulated between H hybrid and HP1 (female parent G70); 2417 genes were up-regulated, and 1631 genes were down-regulated between H hybrid and HP2 (male parent GDH11); 3164 genes were up-regulated and 2200 genes were down-regulated between mid-parent and H hybrid; and 369 genes were up-regulated and 425 genes were down-regulated in HP1 and HP2, respectively (Fig. 2a and b). We also identified that 1148 genes were up-regulated and 1381 were down-regulated in L hybrid and LP1 (female parent K326), 172 were up-regulated, and 205 were down-regulated in L hybrid and LP2 (male parent GDH11), respectively. 135 genes were up-regulated, and 271 were down-regulated in the middle parent and NH hybrids, whereas 422 genes were up-regulated and 388 genes were down-regulated in LP1 and LP2, respectively (Fig. 2c and d, Additional file 4). H exhibited more differential genes than L, which might be one of the reasons for K^+^ content in H hybrids.

### F1 hybrid demonstrated overdominant gene expression pattern

**Fig. 3:**
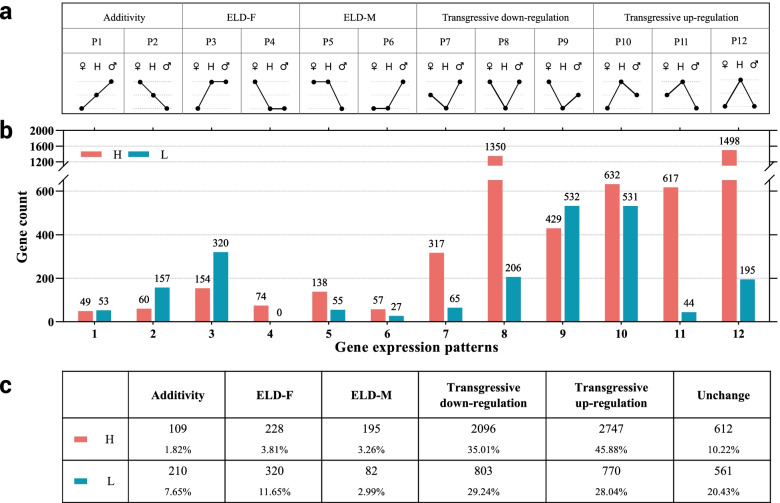
The 12 presumptive additive or non-additive gene expression patterns in F1 hybrid compared to its parents. **a** Expression patterns of 12 types of DEGs. ♂, male parent; H, hybrid; ♀, female parent. **b** the number of genes of each of the 12 expression patterns in H and L hybrids. **c** the number and proportion of five total expression patterns in DEGs

To analyze hybrids and parents, DEGs were divided into 12 expression patterns (P1–P12, Fig. 3a) [40]. Genes in P1 and P2 patterns exhibited additive expression. Genes in P3–P6 patterns were the dominant expression. Genes in the P7-P12 pattern exhibited a transgressive expression, among which P7–P9 were down-regulated overdominant expression, whereas P10–P12 genes were up-regulated over-dominance. Among the overdominant genes, 632, 617, and 1498 genes of H hybrid revealed up-regulated overdominant expression pattern, whereas 317, 1350, and 429 genes demonstrated down-regulated over-dominance expression pattern. Overall, 531, 44, and 195 genes in L hybrid with up-regulation over-dominance expression pattern, and 65, 206, and 532 genes with down-regulation over-dominance expression pattern were present (Fig. 3b). Among the non-additive expression genes (P3–P12), the H hybrid exhibited the highest proportion (80.89%) in the expression pattern of overdominant (P7–P12), and the L hybrid had the highest proportion (57.28%) in the expression pattern of P7–P12 (Fig. 3c). Therefore, our results revealed that the overdominant expression was the primary cause of heterosis of K^+^ content in tobacco leaves.

### Functional enrichment analysis of overdominant genes

**Fig. 4:**
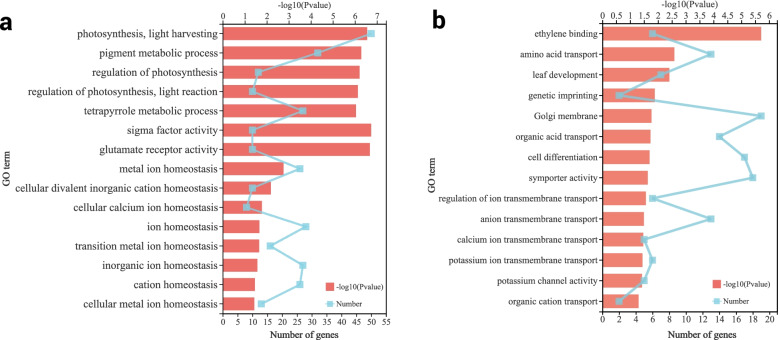
Enriched GO terms for DEGs among *Nicotiana tabacum* L. hybrids and their parents. **a** Enrichment of up-regulated overdominant genes in the H hybrids; **b** Enrichment of non-overdominant genes in the L hybrids

To understand the functions of the overdominant genes, the GO function enrichment analysis of overdominant genes in H hybrid and non-overdominant genes in L hybrid was carried out. As shown in Fig. 4a, the up-regulated overdominant genes in the H hybrid were significantly enriched in photosynthesis, pigment metabolism, tetrapyrrole metabolism, ion homeostasis, cation homeostasis, and other functions (Additional file 5, *P* < 0.05). On the contrary, down-regulated overdominant genes were significantly enriched in ethylene binding, potassium ion transmembrane transport, and potassium channel activity (Fig. 4b, Additional file 6, *P* < 0.05). The non-overdominant genes in the L hybrid were significantly enriched in the potassium ion homeostasis, metal ion homeostasis, calcium ion homeostasis, and potassium chloride transporter activity (Additional file 7, *P* < 0.05).

Interestingly, the up-regulation of overdominant genes in the H hybrid exhibited opposite results. We compared and analyzed the up-regulated overdominant expression gene set of H hybrid and the non-overdominant expression gene set of L hybrid. The results revealed 218 common genes in the up-regulated overdominant expression gene set of H hybrid and the non-overdominant expression gene set of L hybrid, which were significantly enriched in ionic glutamate receptor activity and gated ion channel activity (Additional file 8, *P* < 0.05). These channels have been shown to maintain ion homeostasis in plants [13, 41, 42], proving that the formation of K^+^ content heterosis might be related to the overdominant expression of genes associated with K^+^ homeostasis in hybrid tobacco leaves.

### Overdominant expression of K^+^ homeostasis and transport genes

To analyze the molecular basis of K^+^ heterosis formation, we identified seven core family genes for K^+^ transport and homeostasis in tobacco (Additional file 9). In brief, protein sequences of well-characterized genes of each family from *Arabidopsis thaliana* were used as BLAST query sequences (Additional file 10), and the following sequences in tobacco were filtered by stringent threshold (E-value < 1E-25, % identity > 30 and % query coverage > 30). The genes were identified and verified using a phylogenetic tree (Additional file 11). A total of 26 coding sequences of K^+^ channels and transporters have been identified in the tobacco genome. In tobacco, protein sequences of K^+^ channels and K^+^ transporters exhibited a clear topological relationship, with high bootstrap values (*Solanum lycopersicum* L., *Arabidopsis thaliana*, *Capsicum annuum* L., and *Oryza sativa* Linn.).

**Fig. 5:**
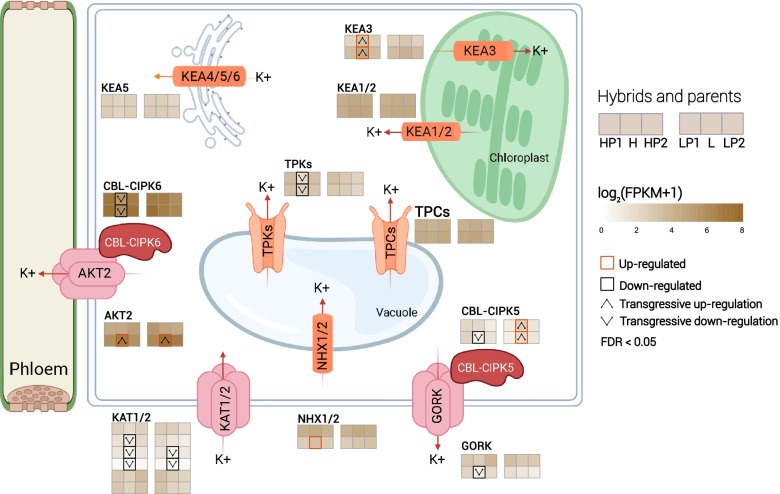
The expression and expression pattern of potassium channel and transport-related genes in H and L hybrids and their parents. Normalized expression values are color-coded according to log2 (FPKM + 1). Significantly (*P* ≤ 0.05) up-regulated and down-regulated genes are indicated by the red box and black box, respectively, and up/down-regulated overdominant expression patterns are indicated by the upper triangle and lower triangle

In leaves (Fig. 5), K^+^ channel genes (*KAT1/2, GORK*, and *AKT2*) demonstrated the down-regulation of overdominant expression in the H hybrid, and the *GORK* activity regulatory complex *CBL-CIPK5* revealed down-regulated overdominant expression in the H hybrids and up-regulated overdominant expression in L hybrids. *AKT2* activity regulation complex *CBL-CIPK6* was down-regulated in H hybrid in an overdominant expression mode, and tandem pore K + channels *TPKs* down-regulated the overdominant expression in H hybrid, and the expression of *TPCs* in H and L hybrid was equivalent to that of their parents. The vacuole K^+^ transporter protein *NHXs* was up-regulated in the H hybrid.

### qRT-PCR analysis

**Fig. 6:**
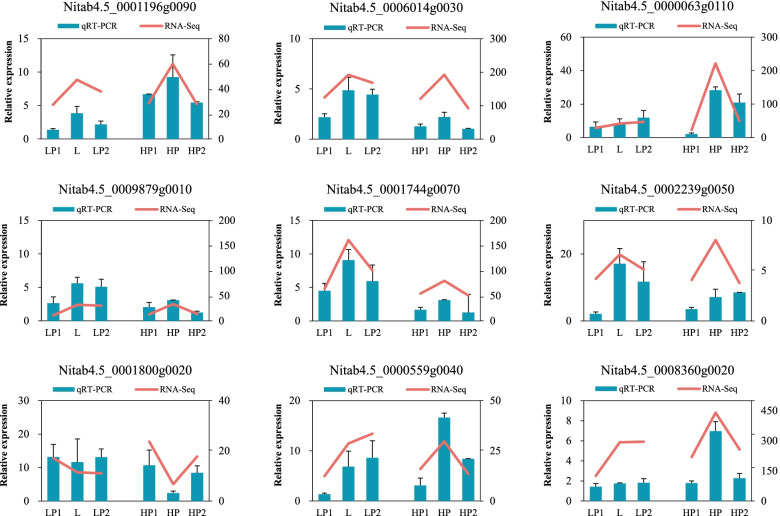
Comparison of gene expression levels of RNA-seq and qRT-PCR in two hybrids and their parents

We randomly selected 9 DEGs for qRT-PCR analysis to verify the DEGs selected by transcriptome. The *Actin* gene was used as the internal reference gene to standardize the expression level of each gene. Additional file 12 lists the genes and corresponding primers used in qPCR. The results illustrated that (Fig. 6), qRT-PCR revealed consistent expression trends with RNA-seq sequencing, although the expression ploidy was slightly different, so the RNA-seq data were reliable (Additional file 13).

## Discussion

K^+^ is highly mobile in plants, and part of potassium in plants is transported back to roots through the phloem. Since K^+^ is bi-directional in the plant plasma membrane, K^+^ spills out through the root system, resulting in lower K^+^ content in tobacco leaves [43]. In our study, The K^+^ content of H hybrid parents revealed a rapid downward trend from day70 to 80. However, the K^+^ content of the H hybrid demonstrated a slow upward trend. On the contrary, the K^+^ content of the L hybrid and its parents decreased rapidly, leading to lower heterosis. Transcription results revealed different expressions of K^+^ transport and homeostasis-related genes in tobacco hybrids (Fig. 4). Briefly, key genes involved in K^+^ inflow and outflow were down-regulated in the H hybrid, indicating that K^+^ flow between cells of the H hybrid was reduced compared with that of parents. Compared with the L hybrid, K^+^ outflow decreased, thus forming heterosis.

The Vacuole is a K^+^ pool. Tandem pore channels *TPKs* and *TPCs* participate in the efflux of vesicular K^+^ from the vesicle into the cytoplasm and play a crucial role in maintaining K^+^ homeostasis. Previous studies have shown that Over-expression of *TPKs* alters K^+^ homeostasis in the vesicle [44], and the *NHXs* control the influx of K^+^ into vacuoles and the homeostasis of K^+^ [45]. Our study revealed that the *TPKs* down-regulate expression in H hybrids, and with the up-regulation of *NHX1/2* expression, the heterozygous advantage of K^+^ content in the leaves of H hybrids developed due to the influx and storage of more K^+^ in the vesicles. At the same time, the K^+^ in the vesicles was more stable than that of their parents, thus promoting a stable and slow increase in K^+^ content of the tobacco leaves of the H hybrids, consistent with the change trend of potassium content in tobacco leaves.

K^+^ channel can be divided into two parts: the channel region, which selects and allows potassium ions to pass through, and the gated switch, which is controlled by the signals in the environment. In our study, the common gene set of up-regulated overdominant expression in H hybrid and non-overdominant expression in L hybrid is significantly enriched in gated ion channel activity and ionic glutamate receptor activity. These channels are involved in cation uptakes, such as Na^+^, K^+^, and Ca^2+^, and play critical roles in pathways related to cellular ion homeostasis and development [46, 47]. However, how these gated ion channels maintain K^+^ homeostasis in tobacco leaves and then regulate heterosis of K^+^ content remains undiscovered.

K^+^ conducts a vital role in photosynthesis and determines the yield and quality of crops [10, 48, 49]. *KEA3*, as a H/K antiporter protein located in the thylakoid membrane, regulates photosynthesis and photosynthetic acclimation [49, 50]. *KEA3* is considered the vital process for high photosynthetic efficiency and CO_2_ assimilation. Our study discovered that *KEA3* up-regulated the overdominant expression in H hybrids. GO enrichment of overdominant up-regulated genes of H hybrid resulted in significant enrichment of photosynthesis, photoresponse regulation, and chloroplast thylakoid membrane. These results indicate that up-regulation of overdominant expressed *KEA3* might improve the photosynthetic efficiency of tobacco leaves, increasing the biomass and yield of tobacco leaves [51, 52]. The biomass of the H hybrid exhibits 8.40% mid-parent heterosis (Additional file 14). However, how *KEA3* and K^+^ regulate heterosis of tobacco biomass must be further explored.

## Conclusions

The present study confirmed the heterosis of the K^+^ content of the hybrids through field trials. The comparative transcriptome analysis of hybrids and their parents revealed that heterosis was related to the overall expression pattern of genes. Compared to their parents, the H and L hybrid had 8.61% and 3.95% differential genes, respectively, and 80.89% and 57.28% of these differential genes exhibited an over-dominant expression, respectively. The GO function enrichment of these overdominant expressed genes informed us that photosynthesis, ion homeostasis, and ion transmembrane transport were significantly enriched, which indicated that the heterosis of K^+^ content in the H hybrid might be attributed to the up-regulation of over-dominant expression of intracellular potassium homeostasis gene. In addition, K^+^ is present in the ionic state in all plant organs, and its absorption, transport, and homeostatic regulation are influenced by several transporter genes and proteins, the over-dominant expression of which may explain the heterosis of tobacco hybrids. In summary, the current study provides a new idea for understanding the related mechanism of heterosis formation of K^+^ content in tobacco leaves.

## Methods

### Plant materials, growth conditions and sample preparation

In the current study, 12 samples with different K^+^ contents were selected as parents, and 35 hybrid combinations were produced using the incomplete diallel cross (NC II) method. All materials were provided by Guizhou Key Laboratory of Tobacco Quality Research. We guarantee that the collection of plant material and experimental research and field studies on plants comply with relevant institutional, national, and international guidelines and legislation. Seeds were sown in greenhouses using the floating seedling method, and the seedlings with five true leaves were then transplanted to the field. The experiment was carried out in the tobacco research base of Guizhou University in Yangwu Township, Anshun City, in 2020 and 2021. The field experiment adopted a random block design with three replicates. The block design was as follows: 2 rows in each plot, 15 plants in each row, a total of 30 plants, with a planting distance and space of 110 × 55 cm. The parents were planted on both sides of the hybrid to reduce the test error. Samples were collected every 10 days, 50–90 days after transplanting. The method was as follows: 3 plants were randomly selected from each plot, and 9–11 leaf positions were selected for mixing. Three replicates were taken. Fresh biological samples were frozen with liquid nitrogen and then stored in an ultra-low temperature refrigerator at -80℃. In addition, the collected fresh samples were used for transcriptome sequencing analysis and RT-qPCR. The remaining samples were de-enzymed in an oven at 105℃ for 30 min, then dried to constant weight at 75℃, and the dried leaves were grounded into fine powder for subsequent determination of K^+^ content.

### Determination of K^+^ content

Briefly, 0.5 g of the sample was soaked in 0.5 mol/L diluted hydrochloric acid and filtered. Then, the K^+^ concentration was measured by flame spectrophotometer [53]. The K^+^ content was calculated according to the following formula:

$${\text{K}}^{+}\text{\%}=\frac{\text{C}\times \text{V}}{\text{G}\times {10}^{6}}\times 100$$

Where C refers to the K^+^ concentration of the liquid to be evaluated (ppm) found from the standard curve; V: the volume of the liquid to be measured; G: dry sample weight (g); 10^6^: weight conversion coefficient.

### RNA isolation and sequencing

Total RNA was extracted from the tissue using TRIzol® Reagent (Plant RNA Purification Reagent for plant tissue) according to the manufacturer’s instructions (Invitrogen), and genomic DNA was removed using DNase I (TaKara). Then RNA quality was determined by 2100 Bioanalyser (Agilent) and quantified using the ND-2000 (NanoDrop Technologies). Only high-quality RNA sample (OD260/280 = 1.8 ~ 2.2, OD260/230 ≥ 2.0, RIN ≥ 6.5, 28 S:18 S ≥ 1.0, > 1 µg) was used to construct sequencing library. Paired-end RNA-seq sequencing library was sequenced with the NovaSeq 6000 sequencer (2 × 150 bp read length).

### Transcriptomics data processing and analysis

By preprocessing the raw reads, short sequences with a length < 25 nt and low quality sequences were removed. After preprocessing, the obtained reads were mapped to the Nicotiana tabacum sequenced cultivar K326 genome [54] using the splice-aware mapping tool, Tophat2 [55]. RSEM was used to quantify gene abundances [56]. Essentially, differential expression analysis was performed using the DESeq2 [57], DEGs with |log2FC| ≥ 2 and p ≤ 0.05 were considered to be significantly different expressed genes. In addition, Gene ontology (GO) functional-enrichment analysis performed to identify which DEGs were significantly enriched in GO terms at Bonferroni-corrected *P*-value ≤ 0.05 compared with the whole-transcriptome background. GO functional enrichment were carried out by Goatools [58].

### qRT-PCR validation

For the validation of the transcriptome data, we randomly selected nine DEGs for qRT-PCR (PCR quantitative real-time) analysis. The total RNA used in the sequencing was reversely transcribed to obtain cDNA, which was used as a template to amplify the target genes, and a real-time fluorescence quantitative PCR (RT-qPCR) experiment was conducted. The RNA was reversely transcribed into cDNA using the FastKing reverse transcription kit (Tiangen, China) as per the manual’s instructions. qPCR was performed using the BIO-RAD CFX96 Real-Time qPCR system. The relative expression of each gene was calculated using 2^−△△Ct^ [59].

### Statistical analysis

SPSS 16.0 software was used for statistical analyses. The variance analysis of K^+^ content was carried out using the Duncan’s new multiple range method (*P* < 0.05). Based on the K^+^ content of tobacco leaves, the values of mid-parent heterosis (MPH) were calculated according to the following method, $\text{M}\text{P}\text{H} \left(\text{\%}\right)=\left(\frac{{\text{F}}_{1}-\text{M}\text{P}}{\text{M}\text{P}}\right)\times 100$, where F_1_ represents the value of the first generation of hybrid and MP represents the average value of parents $\left(\frac{{\text{P}}_{1}+{\text{P}}_{2}}{2}\right)$.

## Supplementary Information

**Additional file 1. **Potassium content of all test materials.

**Additional file 2. **Overview of RNA sequencing data of all 18 transcriptomes.

**Additional file 3. **Transcriptome quality control data.

**Additional file 4. **Gene differential expression analysis.

**Additional file 5. **Go enrichment of up-regulated overdominant expression genes in H hybrids.

**Additional file 6. **Go enrichment of down-regulated overdominant expression genes in H hybrids

**Additional file 7. **Go enrichment of non-overdominant expression genes in L hybrids.

**Additional file 8. **Go Enrichment of up-regulated overdominant expression in H hybrid and down-regulated overdominant gene in L hybrid.

**Additional file 9. **Identification of potassium channel and potassium transport related genes in tobacco leaves.

**Additional file 10. **Information of K^+^ channel proteins in selected species for phylogeny reconstruction.

**Additional file 11. **Phylogenetic analysis of potassium channel proteins.

**Additional file 12.** Primers used in this study.

**Additional file 13. **qRT-PCR’s Ct values.

**Additional file 14.** The biomass of H hybrid and parents.

## Data Availability

The whole *Nicotiana tabacum* genome sequence information was obtained from the solgenomics website (https://solgenomics.net/organism/Nicotiana_tabacum/genome), and the website is open to all researchers. The tobacco materials (‘G70’, ‘GDH11’, ‘K326’, ‘K326×GDH11’, ‘G70×GDH11’) used in this study were supplied by Prof. Liu Renxiang of Guizhou University. The datasets supporting the conclusions of this research article have been included in the article and as additional files. The sequencing database of parents and hybrids has been deposited at NCBI under GEO accession number (GSE198555) https://www.ncbi.nlm.nih.gov/geo/query/acc.cgi?acc=GSE198555.

## Abbreviations

DEGs: Differentially expressed genes
GO: Gene Ontology
qRT-PCR: Real-time quantitative PCR
MP: Mid parent
MPH: Mid-parent heterosis
SPSS: Statistical Product and Service Solutions
