# Supplementary material for: Overdominant expression of related genes of ion homeostasis improves K+ content advantage in hybrid tobacco leaves
Source: BMC Plant Biol. 2022 Jul 12;22:335. doi: 10.1186/s12870-022-03719-1 (PMC9277951; doi:10.1186/s12870-022-03719-1)
Supplement: Supplementary file 11 — Additional file 11. Phylogenetic analysis of potassium channel proteins. [file 12870_2022_3719_MOESM11_ESM.docx]

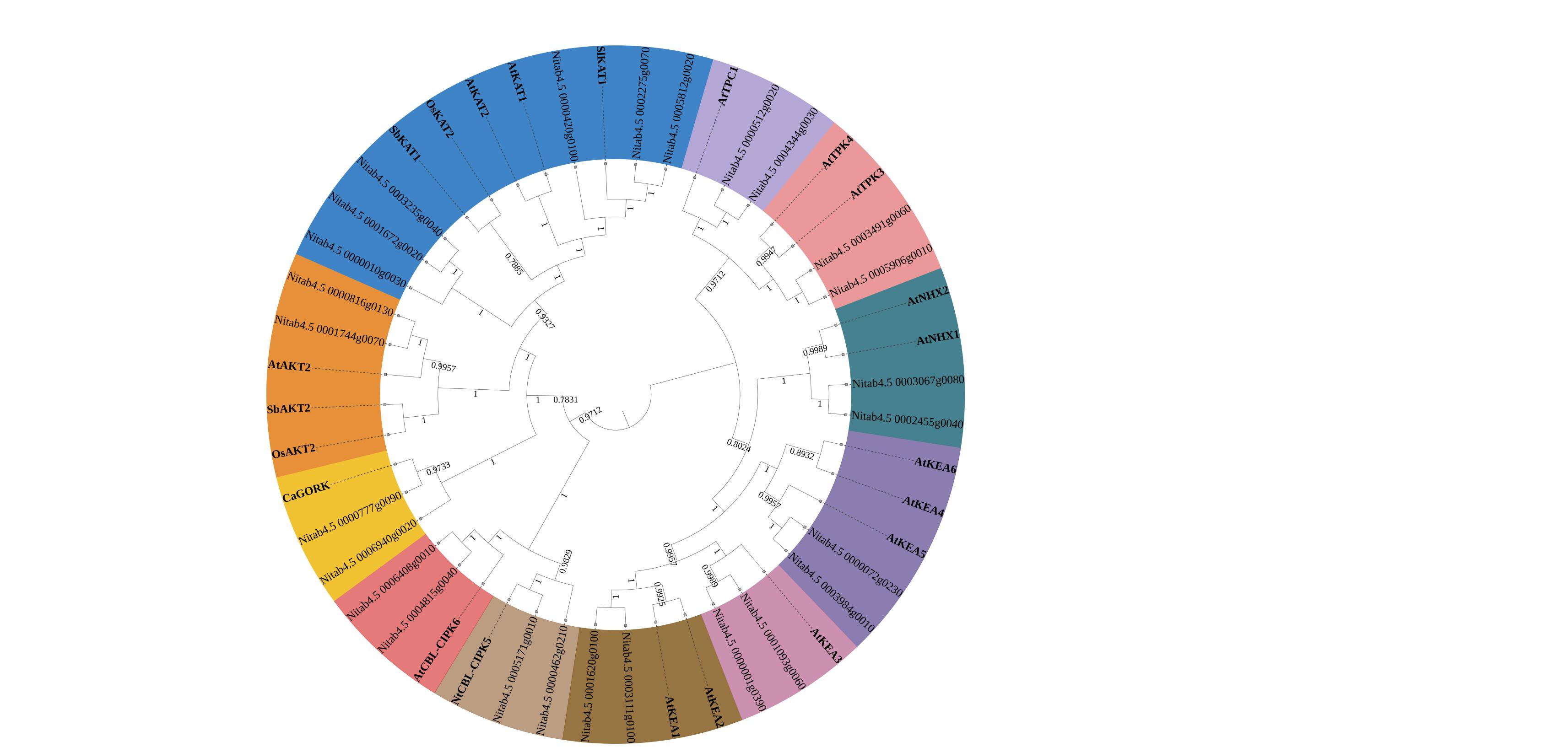


**Fig. S1. Phylogenetic analysis of potassium channel proteins.** Phylogenetic tree was reconstructed by full-length protein sequences of potassium channel proteins, using Bootstrap method as the test of phylogeny and 1000 replicates. The supporting values (bootstrap value) were shown on branches. Proteins in other crops (*Solanum lycopersicum* L., *Arabidopsis thaliana*, *Capsicum annuum* L., and *Oryza sativa* Linn.) are marked in bold.
